# Effect of sonication on particle dispersion, administered dose and metal release of non-functionalized, non-inert metal nanoparticles

**DOI:** 10.1007/s11051-016-3597-5

**Published:** 2016-09-22

**Authors:** Sulena Pradhan, Jonas Hedberg, Eva Blomberg, Susanna Wold, Inger Odnevall Wallinder

**Affiliations:** 1 Division of Surface and Corrosion Science, Department of Chemistry, KTH Royal Institute of Technology, Drottning Kristinas väg 51, 100 44 Stockholm, Sweden; 2 Chemistry, Materials and Surfaces, SP Technical Research Institute of Sweden, P.O. Box 5607, 114 86 Stockholm, Sweden; 3 Division of Applied Physical Chemistry, Department of Chemistry, KTH Royal Institute of Technology, Drottning Kristinas väg 51, 100 44 Stockholm, Sweden

**Keywords:** Sonication, Nanoparticles, Metal release, Particle size, Zeta potential, Copper, Aluminium, Manganese, Dosimetry, DLVO, BSA, Particle dispersion

## Abstract

**Abstract:**

In this study, we elucidate the effect of different sonication techniques to efficiently prepare particle dispersions from selected non-functionalized NPs (Cu, Al, Mn, ZnO), and corresponding consequences on the particle dose, surface charge and release of metals. Probe sonication was shown to be the preferred method for dispersing non-inert, non-functionalized metal NPs (Cu, Mn, Al). However, rapid sedimentation during sonication resulted in differences between the real and the administered doses in the order of 30–80 % when sonicating in 1 and 2.56 g/L NP stock solutions. After sonication, extensive agglomeration of the metal NPs resulted in rapid sedimentation of all particles. DLVO calculations supported these findings, showing the strong van der Waals forces of the metal NPs to result in significant NP agglomeration. Metal release from the metal NPs was slightly increased by increased sonication. The addition of a stabilizing agent (bovine serum albumin) had an accelerating effect on the release of metals in sonicated solutions. For Cu and Mn NPs, the extent of particle dissolution increased from <1.6 to ~5 % after sonication for 15 min. A prolonged sonication time (3–15 min) had negligible effects on the zeta potential of the studied NPs. In all, it is shown that it is of utmost importance to carefully investigate how sonication influences the physico-chemical properties of dispersed metal NPs. This should be considered in nanotoxicology investigations of metal NPs.

**Graphical Abstract:**

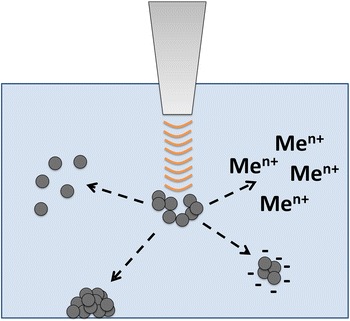

## Introduction

The use of nanoparticles (NPs) in different applications has increased in the last decade (Barkalina et al. 2014; Lines 2008; Prescott and Schwartz 2008). Several driving forces are responsible for this development, including beneficial properties such as large exposed surface areas, different surface properties compared with larger counterparts (Grassian 2008), and tendencies to be mobile. Due to the increased usage of NPs, it is of utmost importance to investigate their potential interactions and fate in the environment and on humans (Hussain et al. 2015; Nel et al. 2009; Oberdörster et al. 2007; Valsami-Jones and Lynch 2015).

The extent of agglomeration and changes in surface properties of NPs in solution is influenced by the method of dispersion. Depending on particle characteristics and selected sonication parameters, suspended NPs will to different extent transform/dissolve, agglomerate and/or interact and form complexes with surrounding medium components (Cohen et al. 2013). This makes understanding of transformation of NPs due to sonication a very important topic in any research field that prepares NP dispersions. Knowledge of the influence of sonication on particle characteristics (e.g. size, surface oxide, zeta potential) and the dissolved fraction is hence essential (Cohen et al. 2015; Hartmann et al. 2015), since this procedure will significantly influence the toxic response of the NPs (Midander et al. 2009; Nel et al. 2015).

Several protocols exist for preparing NP dispersions (Alstrup Jensen et al. 2011; Bonner et al. 2013; Cohen et al. 2014; OECD 2012; Taurozzi et al. 2013; Xia et al. 2013). Typically, these protocols either prescribe a certain delivered acoustic energy to the solution, or a long enough sonication time to ensure that the size of the agglomerates does not decrease with prolonged sonication time (Cohen et al. 2013). Available standard dispersion protocols have been elaborated for NPs of very slow transformation/dissolution rates, such as TiO_2_ or SiO_2_ (Cohen et al. 2015; Hartmann et al. 2015). For example, an acoustic energy of 4.2 × 10^5^ kJ/m^3^ has been reported as the optimal delivered acoustic energy per volume to disperse TiO_2_ NPs (Bihari et al. 2008; Mandzy et al. 2005). Such energy levels may however not be optimal for other kind of NPs and may result in changes in surface characteristics and dissolution properties (Karlsson et al. 2013).

The aim of this work was to gain insights on the influence of sonication and stipulated settings of a standard dispersion protocol (Alstrup Jensen et al. 2014) for preparations of non-inert metallic NP dispersions, including copper (Cu), manganese (Mn), aluminium (Al) NPs and a metal oxide NP (ZnO) for comparison. This paper emphasizes the effect of sonication on the release of metals when preparing particle suspensions, e.g. toxicological studies. This is a different approach compared to other studies that address particle size as a parameter when optimizing the sonication settings (Cohen et al. 2013). If the NPs significantly dissolve during the time frame of sonication, the final suspension will be a complex solution containing both NPs and released metal species rather than the individual NPs (Misra et al. 2012). This is important to consider as, e.g. the toxicological response often depends on particle specifics, chemical speciation of released metal species, or by their combination (Franklin et al. 2007). The release of metals is in this study quantified for dispersions of different particle concentrations and for conditions with different acoustic energies delivered by probe sonication.

This study has used a dispersion protocol based on probe sonication commonly employed in several EU projects related to NPs (Alstrup Jensen et al. 2011; Taurozzi et al. 2012). Other means of dispersion such as ultrasonic bath, vortexing, and manual shaking were also investigated. Ultrapure water was mainly used as the solvent during sonication, as, e.g. biological molecules added as stabilizing agents can be altered and degraded if present in the solvent during the sonication step (Wang et al. 2009). The impact of a stabilizing agent (bovine serum albumin, BSA) on the extent of metal release was elucidated as BSA is recommended in several sonication protocols (Cohen et al. 2015). Sodium perchlorate was used to dilute the NP dispersions in ultrapure water. This is a solution which is fairly non-aggressive towards the metallic NPs. Size distributions, zeta potentials, and extents of metal release were all determined in this solution.

Due to increased collision frequency between the NPs upon sonication, it is expected that higher stock solution concentrations initially result in more agglomeration. To capture most of the concentrations used in stock solutions within the established dispersion protocols (Cohen et al. 2015), particle concentrations of 1 and 2.56 g/L were used.

Prepared NP suspensions are usually considered to be well-dispersed solutions, and the concentration of particles pipetted e.g. to a cell culture plate is assumed to be equal to the nominal concentration of the stock solution. However, if the agglomerates/NPs are dense and heavy, the actual added concentration could be lower than the nominal concentration due to particle sedimentation. To address these aspects, delivered doses of NPs from sonicated stock solutions were investigated for different sonication times.

Calculations based on the Derjaguin, Landau, Verwey and Overbeek (DLVO) theory were performed to estimate the forces between the NPs. This provides insight on mechanisms for agglomeration and enables comparison with the experimental findings.

## Materials and methods

### Nanoparticles

Cu NPs and Al NPs were kindly provided by Assoc. Prof. A. Yu. Godymchuk, Tomsk Polytechnic University, Russia, and were produced by means of wire explosion. The Mn NPs (Lot# 1441393479-680), purity of 99.9 %, were supplied by American Elements (Los Angeles, CA, USA). ZnO standard NPs (NM-110) were supplied by the Joint Research Centre (European Commission, Belgium) and used for comparative reasons.

### Particle characteristics

Particle characteristics of the investigated NPs are compiled in Table 1. More detailed information on the Mn, Cu and Al NPs is given elsewhere (Hedberg et al. 2016b).

**Table 1:** Characteristics of the studied metal NPs

NPs	Mn	Cu	Al	ZnO (Singh et al. 2011)
Primary size (nm)	20 ± 7 (Hedberg et al. 2016b)	100 ± 34 (Hedberg et al. 2016b)	70 ± 26 (Hedberg et al. 2016b)	120 ± 90
Isoelectric point (pH)	3.2 ± 0.6 (Hedberg et al. 2016b)	N/A	6.6 ± 0.7 (Hedberg et al. 2016b)	N/A
Zeta potential (mV)	−24 ± 3 (pH 6.5)	10 ± 2 (pH 5.2)	24 ± 3 (pH 5.2)	−26 ± 4 mV
BET surface area (m^2^/g)	25.5 ± 1.0 (Hedberg et al. 2016b)	7.2 ± 0.7 (Hedberg et al. 2016b)	13.9 ± 0.7 (Hedberg et al. 2016b)	12.4 ± 0.6

### Solutions and chemicals

NaClO_4_ (98 vol%) from Sigma-Aldrich, Sweden, and ultrapure water (18.2 MΩ cm resistivity; Millipore filters, Solna, Sweden) were used to prepare the stock solutions. All experimental equipment was acid-cleaned in 10 vol% HNO_3_ for 24 h and repeatedly rinsed with ultrapure water prior to all experiments. 20-mL Scint-Burk vials (WHEA986581, Wheaton Industries Inc., USA) were used when sonicating the NPs. BSA was purchased from Sigma-Aldrich (Lot # SLBLO253V).

### Sonicator calibration

The calibration of the acoustic energy delivered during sonication was based on an established protocol for NP dispersion (Alstrup Jensen et al. 2014), which in turn is based on a previously published method by Taurozzi et al. (2012). The protocol is divided into two steps. The first step involves a calorimetric method to calibrate the delivered acoustic energy (7056 J) by adjusting the probe sonicator amplitude. This energy was determined by monitoring the temperature increase of a water solution over time, from which the delivered energy is calculated. For the probe used in this study, this resulted in a 20 % sonication amplitude (continuous mode) during 882 s. The second step is to, with given settings, disperse standard silica particles (2.56 g/L, NM200, EU commission, Joint Research Centre) to obtain a particle size distribution between 210 and 270 nm. These sizes were successfully acquired.

### NP dispersion

NPs were weighed in scintillation vials using a microbalance (Mettler- Toledo AG, Model-XP26DR) to obtain stock solutions with particle concentrations of 1 or 2.56 g/L and sonicated (882 or 180 s) in ultrapure water. Dispersions were in addition performed in 0.05 vol% BSA solutions. The BSA was filtered before usage, as described elsewhere (Alstrup Jensen et al. 2011). The glass vials were positioned in an ice-filled bowl with the sonication probe inserted between the upper quarter and upper half of the solution in the vial. NP dispersions from these stock solutions were then added to solutions with NaClO_4_ to reach a final particle concentration of 0.1 g/L. Sonication was performed using a probe sonicator (Branson Sonifier 250, Ø 13 mm, 400 W output power, 20 kHz). Comparative studies were performed using an ultrasonic bath sonicator (Bandelin Sononrex Digitec) and a vortex (Vortex Genie 2).

### Particle size

Particle sizes were analysed with photon cross correlation spectroscopy (PCCS). A Nanophox instrument (Sympatec GmbH, Claustal, Germany) with UVette^®^ cuvettes (routine pack, Sympatec GmbH, Claustal, Germany) was used. Triplicate samples (0.1 g/L) were incubated in an incubator (Merck Cultura Brutschrank Mini Incubator 41 Wärmeschrank) at 25 °C. Measurements were taken after 0, 4 and 24 h, and the size distributions were obtained using the non-negative least squares (NNLS) algorithm. Three independent samples were investigated for each measured time point.

### Metal release

The amount of dissolved metal in solution (metal release) was determined using atomic absorption spectroscopy (AAnalyst 800, PerkinElmer) using the flame (Cu, Mn, Zn) and graphite furnace mode (Al), respectively. Calibration standards for each element were purchased from PerkinElmer (Stockholm, Sweden). The standards were prepared with the following concentrations: 1, 3, 10, 30 mg/L for Mn and Zn; 1, 3, 10, 20, 30 mg/L for Cu; and 15, 30, 100 µg/L for Al. The limits of detection (LOD) were determined to 0.23 (Mn), 0.061 (Cu), 0.020 (Al) and 0.21 mg/L (Zn) based on the method described by Vogelgesang and Hädrich (1998). The recovery of added metal into NaClO_4_, the solution used in metal release experiment, was in an acceptable range for all studied metals (90–100 %).

Standard samples were measured frequently (every 6th sample) for quality control. Recalibration was performed if a drift >5 % was identified. In the graphite mode (for Al), quality controls were made every 5th sample and re-calibration was performed if the deviation exceeded 10 %. Reported release values are average values of triplicates, with blank values subtracted.

To study the amount of NPs (determined as the total metal concentration) transferred to a stock solution, a dose sample was included in each experiment by pipetting 1 mL of stock solution into 9 mL ultrapure water. The samples were exposed at bilinear shaking conditions (Stuart S180 incubator, 12°, 25 cycles/min). To remove non-dissolved NPs, the samples were filtered using an alumina-based membrane with a pore size of 20 nm (Anotop 25, Whatman). Afterwards, the samples were acidified to pH < 2 using 65 vol% HNO_3_. The capacity of the filtration method to separate non-dissolved NPs was verified by parallel studies using ultracentrifugation for 1 h (Beckman Optima L-90K, SW-28 rotor, 52,900 g). The results showed no significant differences in terms of metal concentration (analysed by AAS) between filtered and ultracentrifuged samples.

### Zeta potential

Zeta potential measurements were taken with a Malvern DLS Zetasizer Nano S. The temperature was set to 25 °C, and the sample was left 300 s before measurements in order to stabilize the temperature. Three independent samples were investigated for each exposure condition. The Smoluchowski approximation was used to calculate the zeta potential from the electrophoretic mobility of the NP dispersion. This approximation has some limitations (Bhattacharjee 2016), e.g. the thickness of the electrical double layer has to be much smaller than the particle diameter. The resulting zeta potentials should therefore be interpreted with caution, with more emphasis on the trends rather than on absolute values.

### Theoretical estimations of particle stability in solution

The DLVO (Derjaguin, Landau, Verwey and Overbeek) theory was employed to estimate the stability of the metallic NPs in solution. This theory takes into account attractive van der Waals (vdW) and repulsive electrostatic double-layer (EDL) forces between the particles. In brief, the extent of particle agglomeration is the sum of vdW and EDL forces, i.e. the total interaction force between colloidal particles. The vdW force is always attractive between similar particles. In addition, the vdW interaction is dominated by the properties of the bulk material at large separations, and by the surface layer (e.g. surface oxide) at short separations (Israelachvili 2011).

The non-retarded vdW force between two macroscopic particles can be calculated by using the Hamaker constant, *A*. This constant depends on the chemical properties of particles and applies to any macroscopic geometry. The Hamaker constant is usually calculated using the Lifshitz theory (Tokunaga 2012). The magnitude of the vdW force is determined by the Hamaker constant, i.e. the higher the Hamaker constant, the stronger the vdW force. For conducting materials such as metals with high dielectric properties and refractive indexes, the Hamaker constant should be very high (orders of magnitude larger than for non-conducting materials). This leads to very strong attractive vdW forces between metal particles and, thus, a higher tendency of agglomeration.

The studied metal NPs of this study consist of a solid metal core with a surface oxide (Hedberg et al. 2016b). The effective Hamaker constant was calculated using Eq. 1 assuming a three-layered system: (1) the bulk metal core, (2) the surface oxide and (3) the solution (Ninham and Parsegian 1970)

1 $$\frac{F(D)}{R} = - \frac{1}{6}\left( {\frac{{A_{232} }}{{D^{2} }} - \frac{{2A_{123} }}{{(D + T)^{2} }} + \frac{{A_{121} }}{{(D + 2T)^{2} }}} \right) = - \frac{{A_{\text{eff}} (D)}}{{6D^{2} }}$$

where *A*_232_ = the Hamaker constant: surface oxide/solution/surface oxide, *A*_123_ = the Hamaker constant: metal/surface oxide/solution, *A*_121_ = the Hamaker constant for metal/surface oxide/metal, *A*_eff_ = the effective Hamaker constant, *D* = the distance between particles (nm), *T* = the surface oxide thickness (nm), *R* = particle radius (nm), *F* = force (mN/m).

As expected, the vdW interaction is dominated by the properties of the pure metal at large separations and by the surface oxide layer at short separations. This means that the calculated Hamaker constant is closer to the constant for the pure metal at large separations and to the constant for the surface oxide at short separations.

The EDL force is calculated according to the algorithm of Chan et al. (1980). It uses the nonlinear Poisson–Boltzmann approximation invoking the assumption of interaction at constant charge. The decay length of the double-layer force in monovalent electrolyte solutions is accurately provided by the Debye length (κ^−1^), and the theoretically expected values were used in all calculations, except where noted. The plane of charge and the origin of the vdW force were assumed to lie at the position of the surface oxide.

## Results and discussion

We will first describe differences in resulting particle sizes for commonly used sonication methods to prepare NP suspensions. This will be followed by the specific influence of probe sonication on the extent of particle agglomeration, size distribution, sedimentation, apparent surface charge (zeta potential), release of metals and administered dose.

### Probe sonication is the preferred method for dispersing the non-inert, non-functionalized metal NPs (Cu, Mn, Al)

The Cu NPs were dispersed by different means in ultrapure water followed by immediate dilution in 1 mM NaClO_4_, and thereafter evaluated by DLS. The results are presented in Table 2. Vortexing and manual shaking resulted in particle dispersions that had scattered light intensities in the same order of magnitude as the background (noise) level, i.e. very few NPs in solution. Ultrasonic bath sonication resulted in particle dispersions that gave rise to relatively large scattered light intensities up to 4 h of immersion. This is explained by the fact that large agglomerates scatter proportionally more light than their smaller counterparts. From this followed a rapid sedimentation of these large agglomerates as seen from low scattered light intensities observed beyond the 4-h time point. For the probe-sonicated dispersions, the Cu NPs were smaller and remained longer in solution.

**Table 2:** Comparative study based on scattered light intensities of particle suspensions of Cu NPs using PCCS to assess differences between different methods to prepare metallic NP dispersions

Time after preparation	Scattered light intensity (kcounts/s)			Mean particle size (nm)		
	5 min	4 h	24 h	5 min	4 h	24 h
Manual shaking	nd	nd	nd	nd	nd	nd
Vortexing	nd	nd	nd	nd	nd	nd
Bath sonication	210 ± 43	742 ± 56	nd	1688 ± 64	nd	nd
Probe sonication	701 ± 67	311 ± 47	155 ± 32	494 ± 58	339 ± 32	230 ± 28

The methods were manual shaking (2 min), vortexing (1 min), ultrasonic bath sonication (15 min) and probe sonication (15 min). 1 g/L Cu NPs was dispersed and sonicated in a stock solution of ultrapure water and then diluted to 0.1 g/L in 1 mM NaClO_4_. Delivered acoustic energy with the probe was 1.18 × 10^6^ J/L. Error ranges represent one standard deviation from three independent measurements. nd indicates that the scattered light intensity did not exceed the noise (background) level

**Fig. 1:**
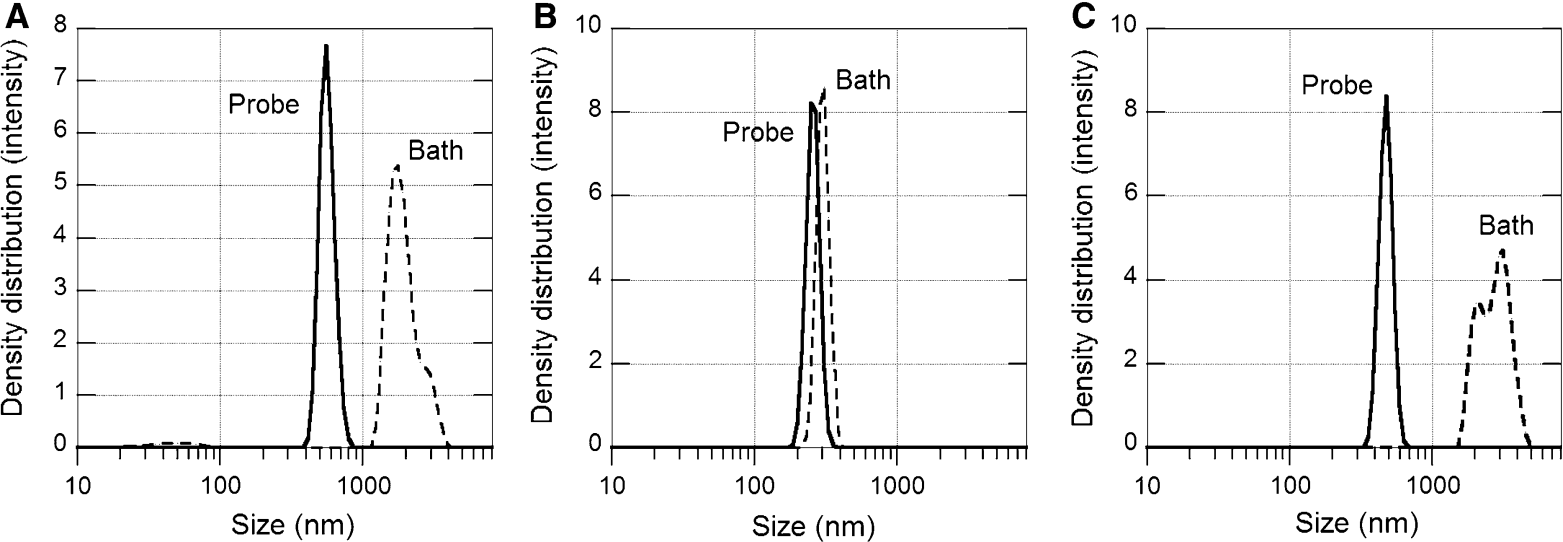
Differences in particle size distribution for particle suspensions of the metal NPs prepared via bath and tip sonication: Cu NPs (**a**), Mn NPs (**b**), Al NPs (**c**). The dispersions were prepared by 15 min sonication in ultrapure water (1 g/L NPs). This was followed by dilution in 1 mM NaClO_4_, resulting in 0.1 g/L NPs. The measurements were taken directly after sonication (~5 min)

The results in Fig. 1 illustrate a comparison between observed particle size distributions in particle suspensions prepared via probe and ultrasonic bath sonication. For the Al NPs and Cu NPs, probe sonication clearly disintegrates particle agglomerates into smaller and more monodisperse units as judged from DLS measurements compared with the bath sonication procedure. The scattered light intensities for Cu and Al indicate more particles in solution when probe sonication is used compared with conditions using bath sonication. However, for the Mn NPs, no clear effect was observed with a fairly similar particle size distribution observed for both sonication methods.

In all, probe sonication resulted in the most homogenously sized particle dispersions. These findings are in line with previous observations (Nickel et al. 2014) and motivate the choice of probe sonication to prepare particle dispersions of the metallic NPs of this study.

### A prolonged probe sonication time results in relatively smaller particle agglomerates, slightly increased metal dissolution and no significant effect on the zeta potential

It was not possible to extract a size distribution from the DLS data for the stock solution of the highest particle concentration (2.56 g/L) due to significant NP sedimentation and very polydisperse particle size distributions. These findings are expected since this very high particle dose leads to a higher collision frequency of the NPs, and hence a higher probability of agglomeration. Similar trends have been reported for NPs of TiO_2_ (Tantra et al. 2015). The effect of sonication time on the particle size distribution will therefore be presented for stock solutions with particle concentrations of 1 g/L.

**Fig. 2:**
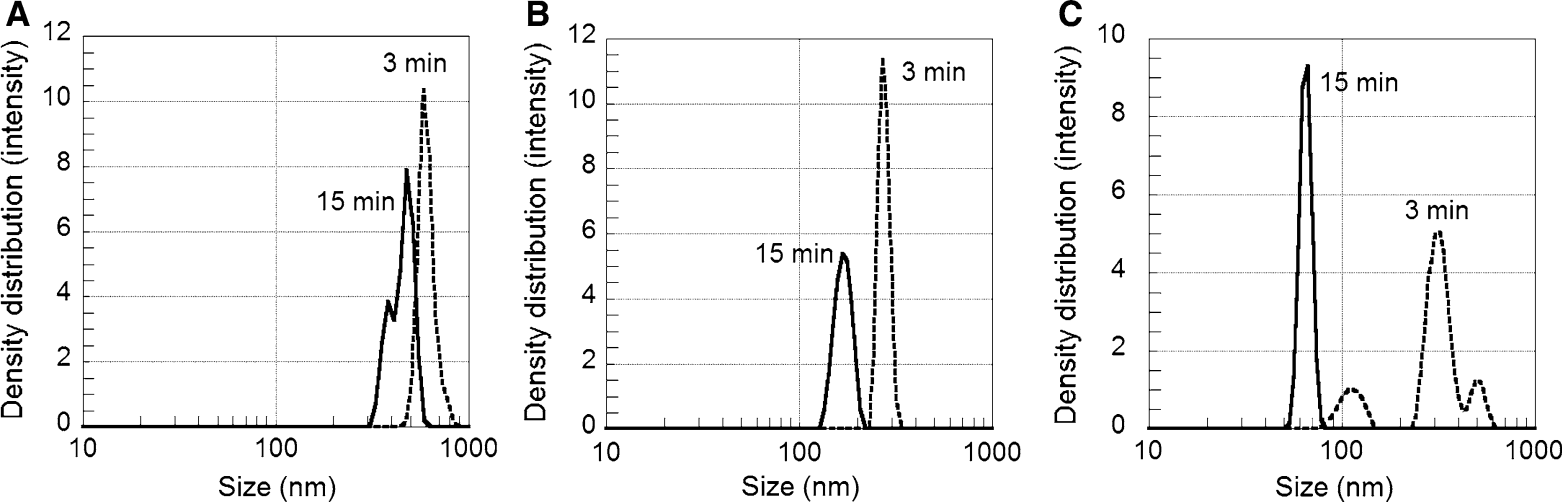
Influence of sonication time on the particle size distribution in solution as deduced by PCCS in 1 mM NaClO_4_ containing 0.1 g/L NPs (diluted from a 1 g/L stock solution) for Cu NPs (**a**), Mn NPs (**b**) and Al NPs (**c**). The measurements were taken directly after sonication (~5 min)

The extension of the sonication time (delivered acoustic energy) from 3 (180 s) to 15 min (882 s) resulted in the disintegration of larger agglomerates into units of smaller size for the Cu, Mn and Al NPs and is shown in Fig. 2. This is expected since more agglomerates are generally disintegrated during prolonged sonication (Cohen et al. 2013; Taurozzi et al. 2011). The reduction in size of the agglomerates was more pronounced for the Al NPs compared with the Cu and Mn NPs. The results reflect that intrinsic particle characteristics largely govern the behaviour of NPs in solution and that the same delivered acoustic energy influences their ability to agglomerate in different material-specific ways. These particle characteristics include, e.g. the isoelectric point, IEP (see Table 1), the apparent surface charge and surface oxide characteristics. For example, the formation of relatively large Cu NP agglomerates is influenced by the fact that the zeta potential of the Cu NPs has the lowest magnitude of the studied NPs.

**Fig. 3:**
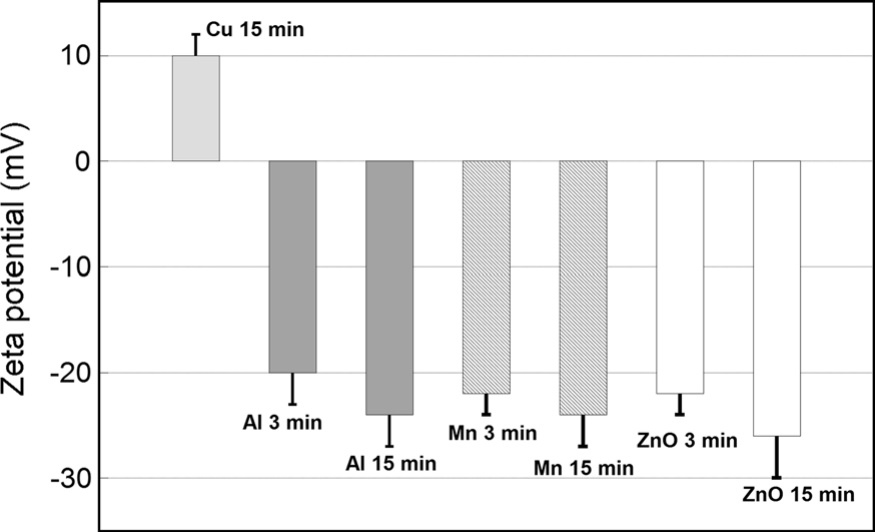
Zeta potential of NPs of Cu, Al, Mn and ZnO in 1 mM NaClO_4_ (0.1 g/L NPs), measured 5 min after probe sonication of the stock solution (1 g/L) for different time periods (3 and 15 min)

The effect of prolonged sonication on the surface charge (zeta potential) is displayed in Fig. 3. Prolonging the time of sonication from 3 to 15 min (i.e. an increased delivered acoustic energy) did not induce any significant change in zeta potential of any of the studied NPs. However, changes in zeta potential after sonication of NPs have previously been observed. This was for example observed when comparing bath and probe sonication (Dickson et al. 2012; Roebben et al. 2011), and non-sonicated and probe-sonicated dispersions of Cu and CuZn NPs (Karlsson et al. 2013). These results show that there is no general rule how sonication will influence the zeta potential of the NPs as the effect of sonication is dependent on NP properties, sonication method and solution. It is therefore important to investigate the possible influence of sonication on the zeta potential when using different sonication methods and settings.

Only a small fraction (<2 %) of the NP solutions was able to pass through a 20-nm pore size membrane directly after sonication, see Table 3. This fraction represents NPs sized less than 20 nm and released metal species of the sonicated and diluted solution (from 2.56 or 1 to 0.1 g/L). The different investigated sonication times and stock solution concentrations of the NPs did not result in any differences in these fractions.

**Table 3:** Fraction of the NPs (sized less than 20 nm) or released metal species of differently sonicated (3 and 15 min) and diluted (from 2.56 or 1 to 0.1 g/L) solutions

NPs	Fraction <20 nm (%)
Cu	0.3 ± 0.1
Cu (BSA)^a^	4 ± 0.03
Mn	1.6 ± 0.4
Mn (BSA)^a^	3.16 ± 0.13
Al	<0.1
ZnO	0.9 ± 0.1

^a^Only investigated for the 1 g/L stock solution and after 15 min of sonication

Some variations in the fractions passing through the 20 nm filter were observed between the different NPs. This is primarily believed to be a result of different physicochemical properties such as surface composition and reactivity, and hence different transformation/dissolution properties (Hedberg et al. 2016b). The passive properties of the metal NPs are strongly connected to the surface oxide properties, which are reduced in the following order: Al NPs ≫ Mn NPs > Cu NPs (Hedberg et al. 2016b). Consequently, the Al NPs released concentrations of aluminium lower than, or close to the LOD.

Increasing the sonication time had no effect on the fraction passing through the 20 nm filter for the Cu NPs. This is believed to be related to the fact that the saturation concentration for Cu in solution was rapidly reached for the given exposure setting. The saturation concentration was 4 mg/L Cu, as calculated by the Medusa software (Puigdomenech 2001). The total amount of released copper was most likely higher than measured by AAS in solution since the precipitated fraction was not accounted for.

Conversely, the addition of 0.05 vol% BSA to the stock solution during sonication, as recommended in the Nanogenotox sonication protocol (Alstrup Jensen et al. 2011), increased the fraction of the smallest units that were able to pass through the membrane. This is illustrated for the Cu and Mn NPs in Table 3. The results are expected since BSA is known to destabilize the surface oxide of reactive metals, e.g. through ligand exchange, and thus accelerate the metal release process (Hedberg and Odnevall Wallinder 2016). In addition, the copper solubility is much higher in DMEM^+^ compared with NaClO_4_ (Hedberg et al. 2016a; Midander et al. 2009). The use of stabilizing agents can hence be problematic as they will influence properties such as surface passivity and dissolution of the sonicated NPs, in addition to effects of sonication on the stabilizing agents themselves (Taurozzi et al. 2011).

**Fig. 4:**
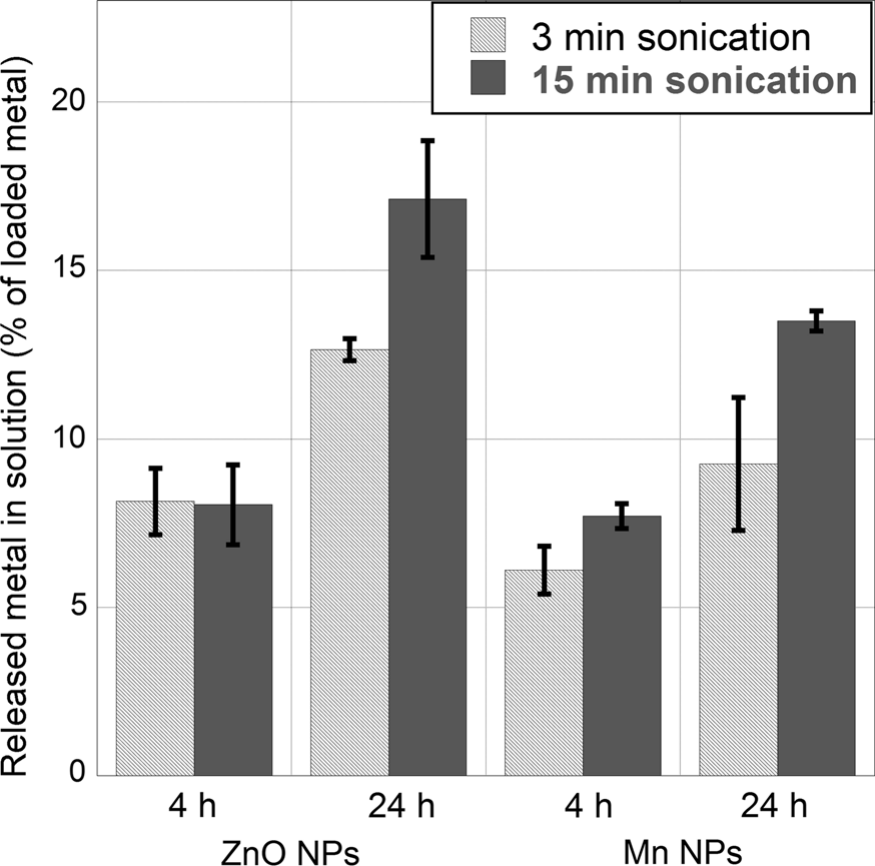
Total amounts of released zinc from ZnO NPs and manganese from Mn NPs in 0.1 g/L 1 mM NaClO_4_ suspensions prepared from probe-sonicated stock solutions (1 g/L) for 3 or 15 min. Data correspond to immersion periods of 4 and 24 h

Small increases in the released amounts of manganese and zinc were observed for the suspensions of Mn and ZnO NPs for prolonged sonication times when the NPs were exposed in 1 mM NaClO_4_ up to 24 h (Fig. 4). For these NPs, the corresponding metal concentrations in solution had not reached the saturation concentrations, as both metals were completely soluble at the given conditions (>100 mg/L solubility). 13 % of the mass of the ZnO NPs was dissolved after 24 h for the stock solution sonicated for 15 min and ~8 % after sonication for 3 min. Corresponding numbers were 16 and 12 %, for the Mn NPs. An increased metal release is expected since the smaller agglomerates of the dispersions sonicated 15 min (Fig. 1) will have a larger specific surface area, which in general result in a higher amount of released metals.

### Theoretical calculations show that strong van der Waals forces result in rapid agglomeration of the metal NPs in solution, which results in rapid sedimentation

DLVO theory calculations were performed to investigate the importance of the electrostatic double-layer interaction (EDL) and van der Waal interactions on the agglomeration behaviour of the metal NPs in solution. Calculations were made for exposures in 1 mM NaClO_4_ using input data on measured surface potentials (zeta potentials), given in Table 1, and on available literature data on Hamaker constants, Table 4 (Russel et al. 1989). The main components of the surface oxide of the Cu and the Mn NPs, CuO and MnO_2_ (Hedberg et al. 2016b; Midander et al. 2009) were considered as the only surface components in the calculations due to the lack of data on Hamaker constants for Cu_2_O and Mn_2_O_3_. These assumptions are believed to not influence the general conclusions as the order of magnitude of their Hamaker constants is assumed to be similar to the main oxide components.

**Table 4:** Hamaker constants for the metal particles and surface oxides used in the DLVO calculations

Material	Hamaker constant (10^−20^ J)
Cu	28.4
CuO	2
Mn	22.6
MnO_2_	7.84
Al	15.4
Al_2_O_3_	3.67
ZnO	1.89

Hamaker constants for the metal oxides are calculated effective Hamaker constants as described in Russel et al. (1989)

Calculations were performed to assess whether an increased surface oxide thickness would change the importance of the van der Waal forces. As expected, the van der Waals interaction is dominated by the properties of the bulk (i.e. the bulk of the metal particle) at large separations and by the surface oxide layer at short separations. The calculations show that the oxide thickness needs to exceed approximately 5 (Cu and Mn NPs) and 2 nm (Al NPs) in order to slightly reduce the strong van der Waals forces. However, this reduction is small as the Hamaker constants of the metal oxides are very similar to corresponding constants for the bare metals. DLVO calculations were therefore performed using the Hamaker constants for the respective oxides, i.e. without considering a core–shell geometry (effective Hamaker constants).

**Fig. 5:**
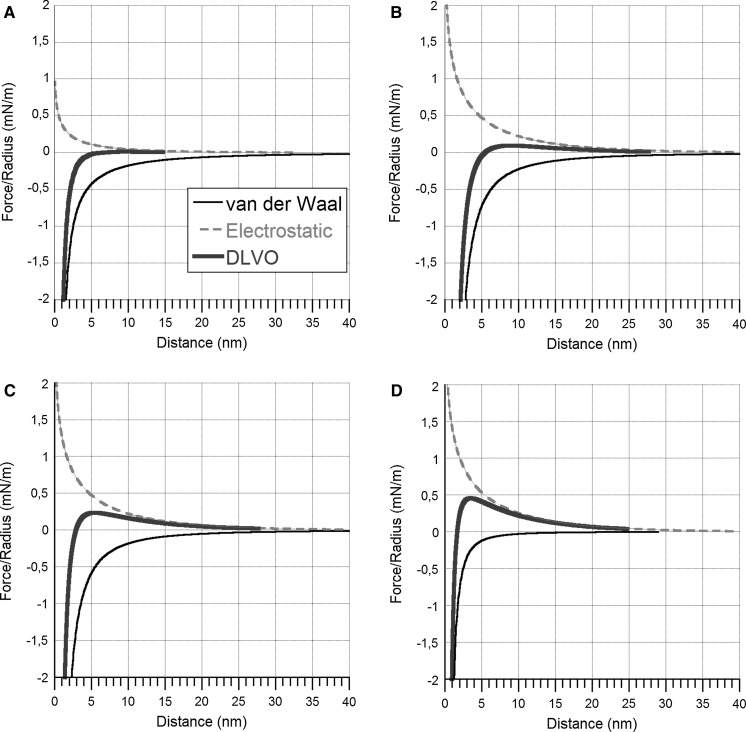
Calculated double-layer force normalized by the particle radius as a function of surface separation for metal NPs of Cu (**a**): *dashed red line* = electrostatic DL force for 10 mV, *black line* = vdW force using *A*_eff_ calculated for Cu and 5-nm-thick CuO film, *blue line* = DLVO force (el.stat + *v*d*W*) calculated using A for CuO (2 × 10^−20^ J). Mn (**b**): *dashed red line* = electrostatic DL force for −24 mV, *black line* = *v*d*W* force using *A*_eff_ calculated for Mn and 5-nm-thick MnO_2_ film, *blue line* = DLVO force (el.stat + *v*d*W*) calculated using *A* for MnO_2_ (7.84 × 10^−20^ J). Al (**c**): *dashed red line* = electrostatic DL force for 24 mV, *black line* = vDW force using *A*_eff_ calculated for Al and 2-nm-thick Al_2_O_3_ film, *blue line* = DLVO force (el.stat + *v*d*W*) calculated using *A* for Al_2_O_3_ (3.67 × 10^−20^ J). ZnO (**d**): *dashed red line* = electrostatic DL force for −26 mV, *black line* = *v*d*W* force calculated using *A* for ZnO (1.89 × 10^−20^ J), *blue line* = DLVO force (el.stat + *v*d*W*). (Color figure online)

The results are illustrated in Fig. 5 for the metal NPs (Cu, Mn, Al) and for ZnO. The magnitude of the repulsive EDL forces was very low for all metal NPs due to the low apparent surface charge. The vdW forces were however very strong due to the high Hamaker constants for the NPs of the metals and their surface oxides. This means that the vdW forces dominate the interaction and result in rapid agglomeration of these NPs in suspension to an extent that causes sedimentation. These theoretical deliberations were consistent with the experimental findings; see, e.g. Table 2 and Fig. 2. The same trend was also observed for the ZnO NPs, although the magnitude of the electrostatic repulsion was somewhat higher due to a higher surface charge (zeta potential), as shown in Fig. 3. However, the repulsive EDL force was not sufficiently high to prevent agglomeration.

### Rapid sedimentation of the metallic NPs results in significant differences between the nominal and the administered particle concentration (dose)

Agglomeration and sedimentation of metal NPs rapidly take place upon suspension preparation, as indicated by the comparative study previously shown for Cu NPs, Table 2, and known from other investigations (Cohen et al. 2015). To investigate the importance of these processes on the administered dose (transferred particle concentration), studies were performed on stock solutions of different particle suspension concentrations (1 and 2.56 g/L). The selection of these high particle concentrations was justified from stipulated levels in established protocols for preparation of NP dispersions (Alstrup Jensen et al. 2014; Cohen et al. 2014). The NPs were dispersed by means of probe sonication for different time periods (~3 and 15 min), equivalent to different amounts of delivered acoustic energy (~2.42 × 10^5^ and 1.18 × 10^6^ J/L, respectively).

**Fig. 6:**
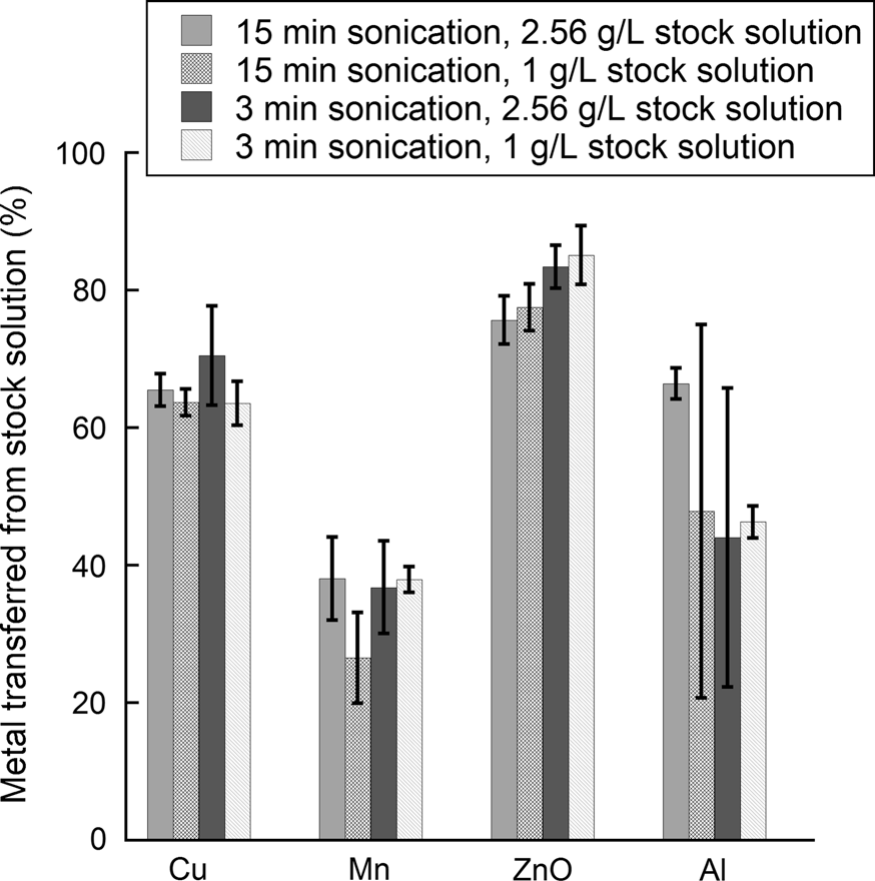
Percentage of administered versus the nominal dose of different metal NPs from stock solution suspensions of different particle concentrations (1, 2.56 g/L) dispersed via probe sonication for different time periods (different delivered acoustic energies; 3 min: 2.42 × 10^5^ J/L and 15 min: 1.18 × 10^6^ J/L)

Differences in the nominal and the administered particle doses (NPs and dissolved species) of the metallic NPs in the two different stock solutions sonicated for different time periods are presented in Fig. 6. The administered dose was for all NPs lower than the nominal dose. Similar trends were evident for both stock solutions with highly material-specific results. The largest difference was observed for Mn NPs (Mn ≥ Al ≥ Cu, ZnO). Similar investigations were performed for a significantly lower particle concentration (0.1 g/L) of the Cu NPs. The same trend was evident, with an administered particle concentration (50 ± 5 %) not statistically different compared with the nominal concentration (Student’s *t* test, *p* < 0.05).

**Fig. 7:**
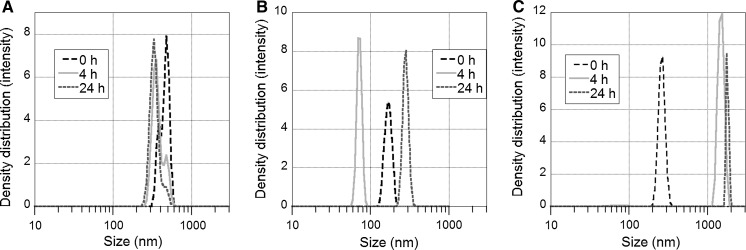
Size distribution measurements with time (0, 4, 24 h) of dispersions of Cu NPs (**a**) Mn NPs (**b**) Al NPs and (**c**) sonicated for 15 min (probe sonication). The measurements were taken in 1 mM NaClO_4_, after dilution of the stock solutions to 0.1 g NPs/L

A lower particle concentration in the administered dose was clearly related to rapid sedimentation of particles from the stock solution, an effect already visible to the naked eye upon sonication. This effect is illustrated in Fig. 7 for the Cu, Mn and Al NPs sonicated for 15 min. For the Cu NPs, the particle size in solution decreased with time, which indicates sedimentation of larger particles (and agglomerates). In the case of the Al NPs, the particle size increased with time, indicative of particle agglomeration. For the Mn NP, the particle size is solution fluctuated over time. This indicates that large agglomerates were present in the solution and occasionally entered the path of the laser beam of the DLS measurement. Observed findings are in agreement with the scientific literature that shows sedimentation to take place within seconds to minutes due to gravitational settling of large agglomerates (tens of µm and beyond; Cohen et al. 2015). The high particle concentrations of the stock solutions (>1 g NPs/L) resulted furthermore in high collision frequencies between the NPs and hence rapid agglomeration. The physicochemical properties governing the intrinsic stability of the NPs influence their tendency of agglomeration, for example the electrostatic and van der Waals forces between NPs discussed previously (Fig. 5).

Below follows some suggestions for reducing the difference between the nominal and the administered dose:

- The addition of a dispersion agent could possibly reduce observed differences; however, such agents change the surface characteristics (adsorbs on the surface) and reactivity of the metal NPs. However, the presence of BSA in solution increased the extent of metal release for the Cu and Mn NPs, Table 3. Studies on Ag NPs by some of the authors show that also the choice of capping agents largely influences both particle stability and the transformation/dissolution properties (Gliga et al. 2014).
- Longer sonication times could perhaps increase the administered dose by making the dispersions more homogeneous and monodisperse (Cohen et al. 2015). However, the reduction in size with prolonged sonication (Fig. 2) did not increase the administered doses (Fig. 6). These results may seem contradictory. However, a significant portion of the particles had already sedimented and were hence not detected in the particle size measurements. Results on the particle size distribution only show agglomerates that remain in solution in a diluted dispersion in which sedimentation is continuously taking place. It seems that the reduction in particle size that takes place for these particles upon prolonged sonication is not sufficient to significantly influence the administered dose. However, a longer sonication time may also result in a larger fraction of dissolved metal species upon exposure over time as illustrated for the ZnO and Mn NPs in Fig. 7.
- Stirring of the stock solution after sonication, while pipetting the administered dose, may improve the dispersion homogeneity. It is though unclear whether rapid sedimentation can be overcome by stirring (not investigated in this study).
- Solution conditions with a pH far from the IEP of given metal NPs may stabilize them in solution (Guiot and Spalla 2012). However, acidic solutions would for most metallic materials (except, e.g. Mo, Si and W) result in a significantly less protective surface oxides and an increased extent of particle transformation/dissolution.

The necessity of measuring the actual dose in each individual case and using freshly prepared dispersions are emphasized in this study. This is due to the fact that the administrated particle doses (measured as total metal) were significantly lower than the nominal doses for all NPs, as shown in Fig. 6, and showed large variation between the different NPs. If not measured, the investigated doses may be highly underestimated from which erroneous conclusions may be drawn and disable comparison with other data.

## Conclusions

In this work, we report on the effect of different sonication methods and sonication parameters on the properties of Cu, Mn, Al and ZnO nanoparticles (NPs). Effects of sonication on agglomeration, metal release, zeta potential and administered dose were elucidated.

This study concludes that agglomeration and sedimentation of the metal NP dispersions rapidly takes place due to strong van der Waals forces, and that these aspects need to be taken into account during preparation of such particle suspensions. Probe sonication is a way forward to disperse such non-inert NPs and reduce the size of formed agglomerates. Nevertheless, rapid sedimentation results in large discrepancies (30–80 %) between the nominal and the administered dose. Probe sonication also influences the extent of metal release, especially when a stabilizing agent is added (BSA). However, small effects were observed when extending the time (delivered energy to the dispersion) of probe sonication. Observed results have large implications on nanotoxicological testing of non-functionalized, non-inert metal NPs.
